# Progress in Medicine

**Published:** 1899-07-22

**Authors:** 


### Progress in Medicine.

### NEUROLOGY.

(*Continued from page* 263.)

**Deep Reflexes.**—In a few cases recorded in medical literature there has occurred the unusual combination of lost knee jerk with presence of ankle-clonus. Mills[8] comes to the following conclusions from examination of this rare syndrome. It may be due to a lesion, such as myelitis, destroying the cord in the region of the patellar reflex arc, *i.e.*, in the second and third lumbar segments; or it may be due to disseminated sclerosis for similar reasons, or to a combination of muscular and neural disease.

Opinions as to the existence of ankle-clonus in cases of hysteria apart from organic disease vary; some think it may occur, others not. Mills agrees with Gowers in thinking that if it occurs it is very rare. There may be a slight excess of myotatic irritability, insufficient to give rise to a true clonus. A spurious foot clonus is common in hysteria, depending on a voluntary contraction of the calf muscles pressing down the foot and varying in degree from time to time. In some few cases of hysterical contracture with spasticity a true ankle-clonus may be present. Excepting these cases, ankle-clonus in hysteria is of the abortive or spurious form.

Achard and Lévi[9] have recorded six cases of tabes without necropsy, and one with necropsy, in which the knee jerks were preserved. The histological examination in the last case showed that the preservation of the knee jerks was due to preservation of the root entrance zones of Westphal in the posterior columns of the lumbar region.

**The Muscle Spindle.**—Batten[10] has recently summarised our knowledge of the muscle spindle, which is a spindle-shaped body occurring in skeletal muscles, abundantly supplied with nerve fibres, which can be traced into the posterior roots, and from which afferent impulses probably arise. It is the sensory nerve termination within the muscle. The proofs that it is sensory in nature are as follows: After section of the anterior nerve roots the corresponding muscle atrophies, leaving the spindle unaltered and its nerve intact. Further, it has an abundant nerve supply far above the number of muscle fibres, the sheath resembles that of other sensory nerve organs, and the nerve fibre is wound in a spiral round the muscle fibre. Again, in acute and chronic disease of the cells of the anterior cornua the muscle atrophies, leaving the muscle spindle intact; and, in tabes dorsalis, changes occur within the spindle. These muscle spindles have been found in nearly all the skeletal muscles, the exceptions being the extrinsic muscles of the tongue, the muscles of the eye, and the diaphragm. They have been found at all ages, from the fourth month of fœtal life onward. They are more frequent in the belly of the muscle than near the tendon; but, in relation to this statement, it is probable that we should recognise in the muscle spindle, the musculo-tendon organ, and the tendon organ variations of the same organ adapting themselves to the tissue in which they are situated. They are, as the name denotes, spindle in shape, from two to four mm. in length. One or more cross-striated muscle-fibres enter one pole of a spindle; as they pass towards the equatorial region they become divided into many smaller ones and lose their striation, and many nuclei appear in their substance and almost fill the fibres. As the fibres pass on towards the opposite pole the nuclei become less numerous, striation reappears, and the fibres join one another again. Correspondingly the number of muscle fibres seen in a cross section varies according to the region of the spindle which is cut across. The nerve supply is abundant; as a rule, at least two nerve bundles enter each spindle, one in the equatorial region and one at the proximal or distal end, the former containing two or three fibres. The sheath of the nerve bundles passes directly into the sheath of the spindle as the nerve enters. The sheath of the spindle consists of a series of laminæ, which give it the appearance of an onion in cross section. The nerve terminations within the spindle are of three varieties—annulo-spiral, flower ending, and the plate ending, the first-named of which is formed by a nerve-fibre wrapping round and round a muscle-fibre about the middle part of its course. In affections of the motor cells and anterior cornua, as already said, no change occurs in the muscle spindle. In a case of tabes Batten found some evidence of degeneration. The entering nerve fibres and the shape of the spindle were unaltered, but the muscle fibres did not stain so well, more especially in the equatorial region.

**Pituitary Body.**—According to Meachen it has been found by Vassale and Sacchi, after removal of the pituitary body in dogs, that the following changes occur. Permanent fall of body temperature, anorexia and listlessness, fibrillary twitchings and tremors of the muscles, periodic attacks of dyspnœa, and rapid progress towards death. It was also observed that many of these symptoms improved when the animals were fed on pituitary extract. Apparently, therefore, the pituitary body produces a secretion which, either by itself or in conjunction with the other ductless glands, is of very great importance in the nutrition of the nervous and muscular systems. But though some lesion of the pituitary gland has been found in every case of acromegaly, the exact connection between the two phenomena is quite unexplained, and the above researches do not shed any light on the subject.

8 Mills, Jour. of Nerv. and Ment. Dis., Mar., 1899. 9 Achard and Lévi, Nouv. Iconographie de la Salpêtrière, 1898. 10 Batten, St. Bart. Hosp. Jour., Mar., 1899. 11 Meachen, Guy's Hosp. Gaz., Feb. 18, 1899.

